# Psychogenic epidemic - mass hysteria phenomena in Portugal

**DOI:** 10.1192/j.eurpsy.2022.999

**Published:** 2022-09-01

**Authors:** A. Costa, S. Jesus, M. Almeida, J. Alcafache

**Affiliations:** Baixo Vouga Hospital Centre - EPE, Psychiatry And Mental Health Department, Aveiro, Portugal

**Keywords:** nocebo effects, mass hysteria, psychogenic ilness

## Abstract

**Introduction:**

Mass hysteria also called mass psychogenic illness (MPI), defined as a social phenomenon, consists of collective anxiety due to a perceived threat and can culminate in a cascade of symptoms suggestive of organic disease without an identifiable cause. Its history dates back to the 14th century and impacts people from all cultures and regions of the world. Before the 20thcentury, MPI emerged across Europe, often in socially isolated convents, in highly stressful environments.

**Objectives:**

The aim of this study is to explore the available literature on mass hysteria phenomena in Portugal, historical origins, applications and eventual position in modern psychiatric semiology.

**Methods:**

Non-systematic review of literature published in Medline/Pubmed. Search terms included: mass hysteria, nocebo, groupthink, emotional contagion.

**Results:**

In Portugal two great phenomena of mass hysteria were described. In 1917, the “sun miracle” occurred, where thousands of individuals reported having seen the sun rotating in the sky and changing its size and colours. Years later, more than 300 students from 14 schools described the same symptoms: dizziness, dyspnea and rash, without an identifiable cause. In common these young people had “sugar strawberries”. In May 2006, the young people in the television series were infected with a vírus, and clinical picture was similar to that presented by young people in real life. For the first time, a fictional illness on television triggered an illness in real life.

**Conclusions:**

More studies should be carried out on these phenomena as their early recognition can have a tremendous impact on the ease of identification, diagnosis and treatment.

**Disclosure:**

No significant relationships.

